# News and notices

**Published:** 2015

## News and notices

### International Society of Geographical and Epidemiological Ophthalmology (ISGEO)

Join ISGEO and get free online access to Ophthalmic Epidemiology. £70 a year. Email **isgeomembers@gmail.com**

### IAPB Africa newsletter

Useful and highly relevant news and updates (including events) related to eye health. For a free subscription, write to Neebha Budhoo: **nbudhoo@iapb.org**

## Courses

### German Jordanian University, Amman, Jordan

Professional diploma and MSc in Vision Rehabilitation. For more information, visit **http://tinyurl.com/rehabcourse** Email: **vtc@gju.edu.jo**

### Community Eye Health Institute, University of Cape Town, South Africa

Short courses, postgraduate diploma, and MPH Community Eye Health. **www.health.uct.ac.za** or email **chervon.vanderross@uct.ac.za**

**Lions Medical Training Centre, Nairobi, Kenya.** Small incision cataract surgery (SICS). Write to: The Training Coordinator, Lions Medical Training Centre, Lions SightFirst Eye Hospital, PO Box 66576-00800, Nairobi, Kenya. Tel: +254 20 418 32 39

### Kilimanjaro Centre for Community Ophthalmology International

Visit **www.kcco.net** or contact Genes Mng'anga **atgenes@kcco.net** and/or **genestz@yahoo.com**

## Stay in touch with us

### Subscriptions


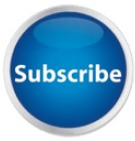
 Contact Anita Shah, ICEH, London School of Hygiene and Tropical Medicine, London WC1E 7HT, UK. **admin@cehjournal.org**

### Visit us online

**www.cehjournal.org**

**www.facebook.com/CEHJournal/**

**https://twitter.com/CEHJournal**

### Write to us


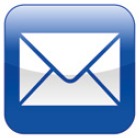
 Share your questions and experiences with us at **correspondence@cehjournal.org** Articles of up to 800 words considered.

### Subscribe to our mailing list

Get an email alert each time a new issue is published! Write to **web@cehjournal.org** or visit **www.cehjournal.org/subscribe**

